# Insights into N-calls of mitochondrial DNA sequencing using MitoChip v2.0

**DOI:** 10.1186/1756-0500-4-426

**Published:** 2011-10-20

**Authors:** Mazin A Zamzami, Gareth R Price, Robert W Taylor, Emma L Blakely, Iulia Oancea, Francis Bowling, John A Duley

**Affiliations:** 1 The University of Queensland, Brisbane, Australia; 2 King Abdulaziz University, Jeddah, Saudi Arabia; 3 Pathology, Mater Health Services, Brisbane, Australia; 4 Mitochondrial Research Group, Institute for Ageing and Health, Newcastle University, UK; 5 Mater Medical Research Institute, Brisbane, Australia

## Abstract

**Background:**

Developments in DNA resequencing microarrays include mitochondrial DNA (mtDNA) sequencing and mutation detection. Failure by the microarray to identify a base, compared to the reference sequence, is designated an 'N-call.' This study re-examined the N-call distribution of mtDNA samples sequenced by the Affymetrix MitoChip v.2.0, based on the hypothesis that N-calls may represent insertions or deletions (indels) in mtDNA.

**Findings:**

We analysed 16 patient mtDNA samples using MitoChip. N-calls by the proprietary GSEQ software were significantly reduced when either of the freeware on-line algorithms ResqMi or sPROFILER was utilized. With sPROFILER, this decrease in N-calls had no effect on the homoplasmic or heteroplasmic mutation levels compared to GSEQ software, but ResqMi produced a significant change in mutation load, as well as producing longer N-cell stretches. For these reasons, further analysis using ResqMi was not attempted. Conventional DNA sequencing of the longer N-calls stretches from sPROFILER revealed 7 insertions and 12 point mutations. Moreover, analysis of single-base N-calls of one mtDNA sample found 3 other point mutations.

**Conclusions:**

Our study is the first to analyse N-calls produced from GSEQ software for the MitoChipv2.0. By narrowing the focus to longer stretches of N-calls revealed by sPROFILER, conventional sequencing was able to identify unique insertions and point mutations. Shorter N-calls also harboured point mutations, but the absence of deletions among N-calls suggests that probe confirmation affects binding and thus N-calling. This study supports the contention that the GSEQ is more capable of assigning bases when used in conjunction with sPROFILER.

## Background

Until recently, studies examining sequence variations within human mitochondrial DNA (mtDNA) have been based on restriction fragment length polymorphisms (RFLP) analysis. With the development of the high-throughput sequencing of PCR products, which is highly sensitive, specific and relatively low cost, it has become more common in the medical and forensic fields to sequence the whole mitochondrial genome [1]. As a result, more rapid sequencing using microarrays-based resequencing is being used increasingly in clinical and research laboratories for identifying mutations throughout the entire mtDNA genome. Resequencing microarrays are a promising technology for diagnostic mitochondrial disorders and related diseases based on changes at the genetics level [2-5]. However, validation of output data particularly for mtDNA, i.e., the accuracy of base identification (or call) for homoplasmic and heteroplasmic mutations, as well as detection insertions or deletions (indels) from these microarrays-based resequencing chips has been a major issue [2,6,7].

The Affymetrix second-generation human mitochondrial resequencing microarray (MitoChip v2.0) was released in 2006. In addition to sequencing the entire mitochondrial genome including the noncoding region (D-loop), it also contains redundant tiling of sequences for 500 of the most common haplotypes including single-nucleotide changes. Each mitochondrial gene of interest is represented by sets of oligonucleotide probes with a matching sequence as reported in MITOMAP: A Human Mitochondrial Genome Database, http://www.mitomap.org[3,4,6]. The MitoChip v2.0 software (GSEQ) assigns a base call at any given position by using the International Union of Pure and Applied Chemistry codes (IUPAC codes), and then compares the base call to the Cambridge Reference Sequence (rCRS). A base call will be assigned either as a wildtype (WT), homoplasmic sequence variant (the presence of a point mutation within all of the mtDNA copies), heteroplasmic sequence variant (the presence of a mixture of more than one type of mtDNA copies some are wildtype and some with point mutation or both copies are point mutations, i.e., both differing from rCRS), or N-call [8].

Failure of the MitoChip v2.0 software to assign a base to any position (N-call) can be for several reasons. In three independent studies, it was concluded that despite improvements in call rate and accuracy achieved by adjusting the GSEQ software parameters, the call rates remain less accurate and less sensitive than conventional dye-terminator sequencing. This failure was attributed to poor hybridization at regions with ≥4 sequential C bases and poor probe performance, [9] as well as variability of the individual target sequence [6,10]. The GSEQ software may also return N-calls and even false-positives when poor hybridization resulting from deletion are present in the sample mtDNA [9]. Balanced against these deficits, the MitoChip still provides a high-throughput tool that is fast, easily automated, and cost-effective for targeting regions of mtDNA sequence variation, with reasonable accuracy [8].

We surveyed the literature for publications relating to re-analysing data generated by resequencing microarrays and specifically for insights into N-call analysis derived from resequencing data. One study used a novel DNA array (Birmingham ReseqUencing Microarray version 1-BRUM1) designed to analyse 92 nuclear DNA genes (involved in metabolic pathways) using specific probes to detect point mutations or known indels, in addition to sequencing. While most deletions (7/10) were not detected, the three samples with homozygous deletions were detected as 'stretches' of N-calls. Furthermore, no obvious reduction in signal intensity was observed at the deletion sites [7]. In a second study, a new software algorithm was developed, ResqMi, that used intensity comparisons to enhance the call rates of resequencing microarray data [11]. Three different resequencing microarrays were analysed using ResqMi: 'CFTR' (targeting the human CFTR gene), 'SARS' (for the Corona virus causing Severe Acute Respiratory Syndrome), and 'Mito' (which became the Affymetrix MitoChip v.1.0). This software succeeded in resolving up to 10% of the N-calls on all three array types using GSEQ as the primary software. In a third study, another software algorithm, sPROFILER, succeeded in resolving more than 80% of N-calls from GSEQ software and allowed 99.6% of their resequencing array to be assigned a base call. An interesting observation in this study was the detection of a continuous stretch of 13 N-calls and a variant call that was attributable to a series of deleted bases in the *TMPRSS3 *gene [2].

For a project we were conducting to examine mutations in mtDNA, we were particularly interested in the detection of indels. As the MitoChip's GSEQ software has no provision for detecting these, we compared the analysis of our mtDNA N-call distribution in the rCRS section using GSEQ, ResqMi and sPROFILER software, based on the hypothesis that N-calls may include indels in mtDNA. Furthermore, it has been reported that depending upon the sequence context, point mutations generally produce the weakest inhibition of probe annealing (least likely to produce an N-call), followed by deletions then insertions [12]. Thus, when searching for indels we expected the highest success would come from examining longer N-call stretches in a sample.

## Materials and methods

### Patient samples

Total DNA was extracted from 16 patient samples, comprising 8 peripheral blood/bone marrow samples and 8 fibroblasts cultures. Blood or bone marrow samples were from 7 patients with myelodyplastic syndrome (MDS) and one patient with chronic lymphoblastic leukemia (CLL), obtained from the Australasian Leukaemia and Lymphoma Group Tissue Bank, Princess Alexandra Hospital, Brisbane, Australia, and the Hematology Clinic at Mater Adult Hospital, Brisbane. Low passage fibroblast cultures from 8 pediatric patients with metabolic disorders indicative of mitochondrial dysfunction were obtained from the Department of Pathology (Cytogenetics), Mater Adult Hospital, Brisbane. This study was approved by the institutional Human Ethics Review Committees of The University of Queensland and the Mater and Princess Alexandra Hospitals, Brisbane, Australia.

### Automated Batch Analysis of Microarray Data

Data analysis of microarrays was carried out using GSEQ software. The Affymetrix MitoChip v.2.0 is tiled with 25-mer DNA probes divided into two sections, the first representing the Cambridge Reference Sequence (rCRS) NC_012920 of mtDNA, while the second section comprises sequences representing 500 of the most common haplotypes recorded in the MitoMap public database http://www.mitomap.org/. These include known mtDNA single nucleotide polymorphisms (SNPs) as well as known pathological mutations or indels. Each base position of mtDNA is represented on the MitoChip by several, tens or hundreds of tiles (i.e., redundant tiling to probe unique or new mutations are repeated many times on the array) [4,8].

The Affymetrix GSEQ algorithm parameter settings recommended to achieve optimal performance were used to analyze the mitochondrial sequences, with 'diploid' selected as the genome model to enable the detection of heteroplasmy. A Quality Score Threshold (QST) of 3 provided the highest performance in terms of overall base calling accuracy and call rates by the software [10]. The GSEQ MitoChip v2.0 microarray software assigns homoplasmic mutations (IUPAC codes A, C, G or T); heteroplasmic mutations (IUPAC codes R, Y, K, M, S, or W), or an N-Call where a base position cannot be assigned by the software.

All microarray (CEL files and CHP files), GSEQ and sPROFILER sequencing data outputs, and all sample information were uploaded to the series records (GSE29550) of Gene Expression Omnibus (GEO).

### sPROFILER and ResqMi software for N call analyses

Data from GSEQ base calls was further analysed using sPROFILER (strand-specific PRObe cell intensity comparison for FILtERing) [2] and ResqMi (REF). sPROFILER is free software designed by Kothiyal *et al *as a novel algorithm developed to improve GSEQ array call rates. It uses MATLAB, a numerical computing and programming language, and is based on intensity signature. When a base cannot be called because of poor hybridization on one of the strands, a threshold is determined by using the next highest intensity ratio on either strand to determine the base call. sPROFILER was not designed to query base calls conforming to the reference sequence (rCRS), as GSEQ is conservative in assigning a base call. Code and detailed description of the sPROFILER algorithm are available at http://www.biomedcentral.com/content/supplementary/1472-6750-10-10-S2.TXT

ResqMi is a base calling algorithm using intensity comparisons and region-wise conformance assessment, to enhance the call rates as well as analysing N-calls produced by microarray gene chips [11]. The algorithm applied two quality measures for filtering any base calls, starting by measuring the conformance (i.e., the fraction of bases equal to the reference in a sliding window on the rCRS), followed by the next highest intensity ratio for each base, to assess signal quality. ResqMi is open source software, available at http://www-ps.informatik.uni-tuebingen.de/resqmi.

### Conventional sequencing of N call stretches

To analyse the nature of N-calls from the sPROFILER output data, the longest N-cell stretch from each of the 16 mtDNA samples was conventionally sequenced, with the 2 following exceptions. For sample mtDNA8 had the 3 longest N-call stretches sequenced, while mtDNA from one fibroblast cell line sample (mtDNA7), which was homozygous for the *94C > A *sequence variant of the nucleotidase gene *ITPA*, was conventionally sequenced across the whole mitochondrial genome, because of other research interests in this gene [13].

For sample mtDNA7, the entire sequence of the mitochondrial genome was amplified, using 36 pairs of M13-tagged oligodeoxynucleotide primers as described previously by Taylor *et al*., 2003 [14]. For the remaining mtDNA samples, the longest stretch of N-calls of each sample was amplified using the appropriate primer for each N-cell stretch, noting that the primers usually extended beyond the ends of the specified stretches. Amplified PCR products were purified (ExoSapIT, GE Healthcare, Buckinghamshire, UK), then sequenced using BigDye^® ^Terminator v3.1 chemistries (Applied Biosystems, UK) on an ABI3130xl Genetic Analyser and directly compared to the revised Cambridge reference sequence (GenBank Accession number NC_012920) using SeqScape software (v2.1.1, Applied Biosystems, UK). The conventional sequencing results were then compared with MitoChip results analysed by GSEQ, ResqMi and sPROFILER.

## Results

Table 1 shows the mean of GSEQ N-calls across the 16 samples; both in the rCRS section of the MitoChip and across the total GeneChip (include the 500 common haplotypes). It can be seen that when samples were further analysed using ResqMi or sPROFILER, there was a striking reduction in the N-call numbers, averaging 35% and 61% decreases respectively for the rCRS section, and 27% and 21% for the haplotypes section, across the 16 samples. These decreases were significant for all samples (P < 0.0003 to < 0.0005), calculated using the Wilcoxon signed rank test.

**Table 1:** N-call analysis of mtDNA sequences using MitoChip v2.0 with Affymetrix GSEQ, sPROFILER, and ResqMi software.

Software	Means of N-Calls in rCRS (n = 16)	N-calls decrease %	P value	Total N-Calls	N-calls decrease %	P value
**GSEQ**	623			7212		

**ResqMi**	407	35	p < 0.0005	5284	27	p < 0.0003

**sPROFILER**	239	61	p < 0.0003	5698	21	p < 0.0003

The results are shown as average of N-Call numbers and % decreases. P value calculated using the Wilcoxon signed rank test comparing the percentage drop in N-call using sPROFILER software and ResqMi software against Affymetrix GSEQ software. Total N-calls refer here to both MitoChip sections (rCRS and haplotypes).

**Figure 1:**
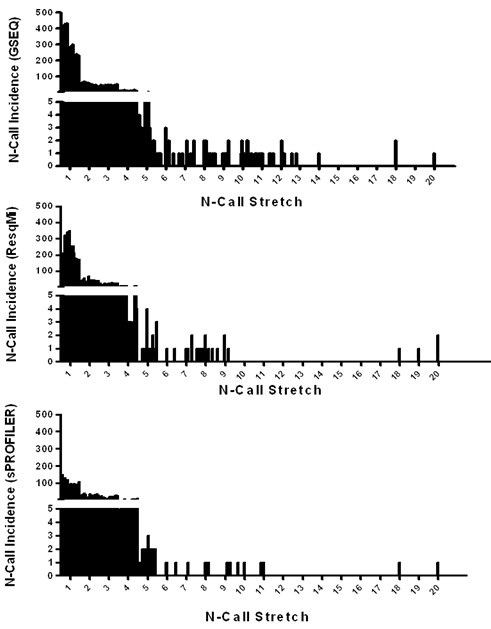
**The incidence of N-call stretches following mtDNA sequencing using MitoChip with GSEQ *vs*. sPROFILER and ResqMi software**.

Figure 1 shows re-analyses of the N-call stretches produced by GSEQ using sPROFILER (See: Additional file 1, Table S1) or ResqMi (See: Additional file 1, Table S2), where approx. 2% of the remaining N-call stretches were ≥5 bases for both re-analyses. Interestingly, the two longest stretches from the GESEQ analysis (e.g. 20 and 18 bases) remained following ResqMi or sPROFILER re-analyses. Visual inspection of Figure 1 suggested that each sample may have hundreds of single base N-calls, but the incidence of N-calls decreased suddenly for N-call stretches of approximately 4 or more bases. We re-analysed the standard deviation of N-call stretches using 'Control Chart' (or Shewhart Chart), a statistical calculation which puts natural process limits on sets of data (See: Additional file 1, Figure S1). Control Chart output found that N-call stretches ≥ 4 bases long were within a limit of 3 standard errors. This indicated N-call stretches < 4 contained high variable data which were less likely to mask genuine underlying biological changes from the reference sequence, i.e. deletions and insertions (indels).

However, reanalysis using sPROFILER appeared to be more effective, as it produced fewer and shorter N-call stretches compared to ResqMi (not shown). Moreover, it was also noticed that, in contrast to GSEQ and sPROFILER, the total number of sequence variants (i.e. the homoplasmic and heteroplasmic mutations) compared to the rCRS greatly increased using the ResqMi software (See: Additional file 1, Table S3). This suggested using the ResqMi may result in base miscalling, particularly increasing heteroplasmic calls. To confirm the accuracy of the new base calls would have required complete conventional sequencing of all samples. For sample mtDNA7, comparison of the ResqMi re-analysis of the MitoChip sequence with complete conventional sequencing showed that all heteroplasmy calls produced by ResqMi in fact matched the rCRS as called by conventional sequencing. We did not use this software for further analyses in this study. Comparison of conventional sequencing with the MitoChip rCRS is discussed below.

Table 2 shows that N-call stretch lengths ≥ 4 decreased after re-analysing GSEQ with sPROFILER. Based on the report that indels may impact on microarray probe annealing, [12] the longest N-call stretch from each sample was sequenced conventionally, with two exceptions (it was not possible to sequence all N-call stretches due to financial constraints). For sample mtDNA8, the 3 longest N-call stretches (10, 18 and 20 bases) were sequenced. For sample mtDNA7, complete sequencing was performed because this was derived from a fibroblast cell line homozygous for the *94C > A *sequence variant of the nucleotidase gene *ITPA *(see Methods).

**Table 2:** Lengths of N-call stretches following mtDNA sequencing using MitoChip with GSEQ and sPROFILER.

Sample Name	N-Call stretch lengths (bp)		Mutation found by conventional sequencing
		
	*GSEQ	*sPROFILER	
**mtDNA1**	5, 6, 9, 13	4, 5	1 point mutation (in 5 base stretch)

**mtDNA2**	5, 6, 12	4, 5, 6	1 point mutation (in 6 base stretch)

**mtDNA3**	5, 6, 7, 11	4, 10	1 point mutation, 1 insertion (in 10 base stretch)

**mtDNA4**	5, 6, 11	4, 5	1 insertion (in 5 base stretch)

**mtDNA5**	5, 13	4, 5	1 insertion (in 5 base stretch)

**mtDNA6**	5, 7, 11	4, 5, 11	1 point mutation, 1 insertion (in 11 base stretch)

**mtDNA7**	5, 10	4	Entire mtDNA sequenced: 1 point mutation (in 4 N-call stretch)

**mtDNA8**	5, 6, 8, 9, 10, 11, 14, 18, 20	4, 5, 6, 8, 10, 18, 20	1 point mutation (in 20 base stretch) 2 point mutations (in 18 base stretch) 1 point mutation (in 10 base stretch)

**mtDNA9**	5, 6, 9, 11, 12	4, 5, 8	2 insertions (in 8 base stretch)

**mtDNA10**	5, 7, 8, 9, 10	4, 5, 7, 9	1 point mutation (in 9 base stretch)

**mtDNA11**	5, 6, 8, 9, 12	4, 5, 8	1 point mutation (in 8 base stretch)

**mtDNA12**	5, 9, 10	4, 9	1 insertion (in 9 base stretch)

**mtDNA13**	5, 7, 8, 9, 10	4, 5, 9	1 point mutation (in 9 base stretch)

**mtDNA14**	5, 8, 10	4, 5	No point mutation or indel (in 5 base stretch)

**mtDNA15**	5, 6, 7, 11	4, 5	No point mutation or indel (in 5 base stretch)

**mtDNA16**	5, 7, 8, 10, 11	4, 6	No point mutation or indel (in 6 base stretch)

*Only stretches ≥ 4 bases identified in GSEQ are listed. For each sample only the longest N-call stretch was sequenced conventionally (except mtDNA7 and mtDNA8). Shorter N-call stretches occurred multiple times-as seen in Figure 1.

In 6 of the N-call stretches sequenced conventionally, we found 7 insertions (i.e. one N-call stretch had 2 insertions). Insertion type m.309insC accounted for 6 of the 7 insertions found within the N-call stretches, while mtDNA9 also had insertion type m.315insC located one base outside its N-call stretch. Conventional sequencing revealed 12 point mutations within the N-call stretches. No deletions were found by conventional sequencing of the N-call stretches. Of 3 N-call stretches that showed no sequences differences compared with the rCRS sequence reference on the MitoChip, 2 were found to be CG-rich.

For the sample mtDNA7, which was completely sequenced (Table 3), one deletion (m.3107delC) was called by MitoChip and confirmed by conventional sequencing: this deletion was acknowledged by Affymetrix as being omitted from the resequencing array at the time of GeneChip design due to an error in the original human mtDNA sequencing [15]-this error has been retained in the rCRS [16]. One deletion found by conventional sequencing, m.15944delT, was called as a 'heteroplasmic substitution' by MitoChip, and 2 other deletions (m.523_524delAC, m.16193delC) were assigned as normal bases by MitoChip. On the other hand, MitoChip did not call any insertions in any samples, but conventional sequencing of mtDNA7 revealed 2 insertions (m.315insC, m.16193insC) (Table 3).

**Table 3:** mtDNA homoplasmic and heteroplasmic mutations identified in fibroblast sample 7 (mtDNA7).

mtDNA Gene Location	Nucleotide Position	Ref	MitoChip call	Nucleotide change		Comment
			**mtDNA7**	**MitoChip GSEQ**	**Conventional sequencing**	

D-loop	146	T	N	T > N	T > C	**N = point mutation **(1)

D-loop	315	C	C	C	insC	**Insertion **(1)

D-loop	464	A	N	A > N	A > C	**N = New point mutation **(1)

D-loop	523-524	AC	AC	AC	delAC	**Deletion **(1)

16S rRNA	1814	A	R	A > G	-	**Heteroplasmic mutation **(2)

**16S rRNA**	**3107**	**C**	**-**	**delC**	**delC**	**Deletion**

MTND2	5264	C	T	C > T	C > T	**New**

MTND4	11466	T	K	T > G	-	**Heteroplasmic mutation **(2)

MTTL2	12307	A	R	A > G	-	**Heteroplasmic mutation **(2)

MTTL2	12309	A	R	A > G	-	**Heteroplasmic mutation **(2)

MTND5	13037	C	M	C > A	-	**Heteroplasmic mutation **(2)

MTTT	15944	T	Y	T > C	delT	**Deletion **(3)

MTTP	15977	C	T	C > T	C > T	**New**

D-loop	16189	T	N	T > N	T > C	**N = point mutation **(1)

D-loop	16192	C	C	C	insC	**Insertion **(1)

D-loop	16193	C	C	C	delC	**Deletion **(1)

D-loop	16343	A	N	A > N	A > G	**N = point mutation **(1)

Homozygous for the *ITPA 94C > A I *sequence variant (*ITPA *^-^/^-^), using MitoChip v2.0 *vs*. conventional sequencing (dye-terminator sequencing). Abbreviations = mitochondrial genes according to international notation (see www.mitomap.com). In IUPAC base codes r = A or G, k = G or T, m = A or C, y = C or T, and n = A, C, G or T. The deletion 3107C was omitted from the resequencing array. (1) not assigned by MitoChip; (2) not assigned by conventional sequencing; (3) assigned by MitoChip as point mutation not as deletion; "-" not detected by conventional sequencing.

Moreover, for the 145 N-call bases remaining after sPROFILER analysis for mtDNA7 (Additional file 1, Table S1), which ranged from a single N-call base to four N-call bases length, conventional sequencing revealed that 4 of these N-call bases to be point mutations (Table 3). One of these point mutations was found among the N-call stretches of 4 bases and the other 3 point mutations were found among single N-calls. The remaining 141 N-calls matched the rCRS. These results correlated with our statistical calculation using "Control Chart" (Additional file 1, Figure S1), which found that N-call stretches < 4 were high variable data: for this reason the shorter N-call stretches in the other samples were excluded from conventional sequencing but these may have contained point mutations or indels.

For mtDNA7, a total of 35 homoplasmic and heteroplasmic mutations were identified using MitoChip, with 30 of these mutations being confirmed by conventional sequencing: 2 new homoplasmic mutations were not detected by Mitomap, while 5 heteroplasmic mutations (m.1814A > G, m.11466T > G, m.12307A > G, m.12309A > G, m.13037C > A) detected by MitoChip were not confirmed by conventional sequencing. Two new homoplasmic substitutions assigned by MitoChip (m.5264C > T and m.15977C > T) were not reported on Mitomap but were confirmed by conventional sequencing. Four single base N-calls assigned by MitoChip were confirmed by conventional sequencing as point mutations, three of them were previously reported by Mitomap (m.146T > C, m.16189T > C, m.16343A > G) and one was a new mutation (m.464A > C).

## Discussion

This project aimed to re-analyse N-call stretches assigned by GSEQ, the Affymetrix MitoChip v2.0 software, using mtDNA isolated from 16 patient samples. A significant reduction in N-calls has been previously reported by the use of sPROFILER or ResqMi software, which both act essentially by choosing the ratio of the highest to the next highest signal intensity on either strand, to improve base calling [2]. However, the accuracy of either sPROFILER or ReseqMi re-analyses of GSEQ N-calls has not been demonstrated previously. Our study confirmed the significant reduction in N-calls using both types of software, but we found that ReseqMi produced a larger number of N-call stretches compared to sPROFILER, although the longest N-call stretches (i.e. 18 and 20 bases) of one sample (mtDNA 8) remained following both ResqMi and sPROFILER analyses. Importantly, although sPROFILER analysis produced a significant decrease in N-calls, in both the rCRS and haplotypes sections of the MitoChip, this did not affect the homoplasmic or heteroplasmic mutation rates compared to Affymetrix GSEQ software, whereas ResqMi analysis significantly increased the mutation rates.

The significant decrease in the MitoChip N-calls using sPROFILER allowed us to narrow down the areas to search for possible point mutations or indels. We then used conventional sequencing of the longer N-call stretches produced by sPROFILER, [2] as well as completely sequencing one sample (mtDNA7). The conventional sequencing found 19 mutations overall, comprising 7 insertions and 12 point mutations. Interestingly, conventional sequencing found no deletions among the N-call stretches examined, but did find insertions.

The failure of the Mitochip to detect deletions (or the software to assign them as N-calls) presumably arises from peculiarities in the conformation of the array probes when binding mtDNA. This corresponds well with a previous microarray study, which reported that insertions had the highest negative impact on probe annealing [12]. On the other hand, both of the longest N-call stretches (18 and 20 bases) contained point mutations (multiple, in the case the 18 base stretch) rather than insertions. Complete conventional sequencing of the sample mtDNA7 showed that 3 out of 145 single-base N-calls in the sample comprised point mutations, of a total of 17 sequence variants found in this sample (Table 3). This one example of complete sequencing thus indicated that shorter N-calls may also hide sequence variants.

With the significant increases of throughput applications for large targeted sequencing or whole mitochondrial genome resequencing the need for new approaches towards data analysis and variant identification, combined with higher sequencing accuracy, will become paramount for both accuracy and to minimise sequencing costs. This is particularly important for variation in mitochondrial DNA from different tissues of the same individual, because of the presence of sequence variants among the large numbers of mtDNA copies within a tissue or even in a single cell [17]. New techniques such as digital sequencing [17] or 'next generation sequencing' [18] may eventually provide more comprehensive sequencing but their use for screening will depend upon low cost and ease/speed of use.

## Conclusions

This small study of mtDNA using MitoChip demonstrated that N-calls stretches can mask important sequence information. Nonetheless, our study showed that using MitoChip and GSEQ software coupled with sPROFILER can provide economical sequence screening of mtDNA, but with limitations, particularly for calling indels. Insertions were assigned as N-calls, but one aspect of the array that still needs further refinement is the inability to detect deletions [6,9,10]. Our study suggested that gains in economy may be achievable by focusing conventional sequencing efforts on longer stretches of N-calls. These stretches were better identified using sPROFILER than ResqMi software, improving the base calling and reducing N-calls to those that frequently represented an undefined change (i.e. a point mutation or insertion) rather than failures of Affymetrix GSEQ software to assign a call [2].

## Competing interests

The authors declare that they have no competing interests.

## Authors' contributions

MAZ is the primary author who designed and conducted the study. GRP co-designed the study and co-wrote the paper. ELB did the conventional sequencing and co-wrote the paper. IO helped with cell lines study and reviewed the paper. RWT co-designed the study and arranged for the conventional sequencing and reviewed the paper. FB co-designed the study and reviewed the paper. JAD was study chair, co-designed the study and co-wrote the paper. JAD and FB are both designated as senior authors. All authors read and approved the final manuscript.

## Supplementary Material

Additional file 1 **N-call analyses and homoplasmy/heteroplasmy in 16 mtDNA samples**. Comprises: Table S1: N-call analysis of mtDNA sequences using MitoChip v2.0 with Affymetrix GSEQ and sPROFILER software. Table S2: N-call analysis of mtDNA sequences using MitoChip v2.0 with Affymetrix GSEQ and ResqMi software. Table S3: Total sequence variants (homoplasmic and heteroplasmic) found in mtDNA sequences compared to rCRS using MitoChip v2.0. Figure S1: Control Chart calculation based on the standard deviation of the N-call stretches. Click here for file
