# Supplementary material for: Insights into N-calls of mitochondrial DNA sequencing using MitoChip v2.0
Source: BMC Res Notes. 2011 Oct 20;4:426. doi: 10.1186/1756-0500-4-426 (PMC3208482; doi:10.1186/1756-0500-4-426)
Supplement: Additional file 1 — N-call analyses and homoplasmy/heteroplasmy in 16 mtDNA samples. Comprises: Table S1: N-call analysis of mtDNA sequences using MitoChip v2.0 with Affymetrix GSEQ and sPROFILER software. Table S2: N-call analysis of mtDNA sequences using MitoChip v2.0 with Affymetrix GSEQ and ResqMi software. Table S3: Total sequence variants (homoplasmic and heteroplasmic) found in mtDNA sequences compared to rCRS using MitoChip v2.0. Figure S1: Control Chart calculation based on the standard deviation of the N-call stretches. [file 1756-0500-4-426-S1.DOCX]

N-call analyses and homoplasmy/heteroplasmy in 16 mtDNA samples

Table S1: N-call analysis of mtDNA sequences using MitoChip v2.0 with Affymetrix GSEQ and sPROFILER software. Sample names refer to DNA extracted from fibroblast (mtDNA1-8) and blood (mtDNA 9-16) samples. The results are shown as N-Call numbers of % decreases. *p < 0.0003 calculated using the Wilcoxon signed rank test comparing the percentage drop in N-call using sPROFILER software against Affymetrix GSEQ software. Total N-calls refer here to both MitoChip sections (rCRS and haplotypes).

| Sample Name | rCRS N-Calls | rCRS N-Calls | N-calls decrease % | Total N-Calls | Total N-Calls | Total N-calls decrease % |
| --- | --- | --- | --- | --- | --- | --- |
|  | GSEQ | sPROFILER |  | GSEQ | sPROFILER |  |
| mtDNA1 | 631 | 226 | 64 | 6636 | 5214 | 21 |
| mtDNA2 | 783 | 315 | 60 | 7044 | 5558 | 21 |
| mtDNA3 | 630 | 244 | 61 | 6724 | 5041 | 25 |
| mtDNA4 | 792 | 286 | 64 | 7277 | 5464 | 25 |
| mtDNA5 | 607 | 232 | 62 | 7019 | 5591 | 20 |
| mtDNA6 | 812 | 227 | 72 | 7413 | 5948 | 20 |
| mtDNA7 | 373 | 145 | 61 | 6508 | 4719 | 27 |
| mtDNA8 | 681 | 116 | 83 | 9787 | 7436 | 24 |
| mtDNA9 | 658 | 278 | 58 | 8218 | 7102 | 14 |
| mtDNA10 | 565 | 236 | 58 | 7335 | 6132 | 16 |
| mtDNA11 | 659 | 198 | 70 | 7496 | 5851 | 22 |
| mtDNA12 | 550 | 244 | 56 | 6582 | 5054 | 23 |
| mtDNA13 | 550 | 245 | 55 | 6898 | 5333 | 23 |
| mtDNA14 | 587 | 261 | 56 | 7275 | 6521 | 10 |
| mtDNA15 | 505 | 277 | 45 | 6466 | 4948 | 23 |
| mtDNA16 | 580 | 287 | 51 | 6718 | 5258 | 22 |
| Mean | 623 | 239 | *61 | 7212 | 5698 | *21 |

**Table S2: N-call analysis of mtDNA sequences using MitoChip v2.0 with Affymetrix GSEQ and ResqMi software.** Sample names refer to DNA extracted from fibroblast (mtDNA1-8) and blood (mtDNA 9-16) samples. The results are shown as N-Call numbers of % decreases. *p < 0.0005 calculated using the Wilcoxon signed rank test comparing the percentage drop in N-call using ResqMi software against Affymetrix GSEQ software. Total N-calls refer here to both MitoChip sections (rCRS and haplotypes).

| Sample Name | rCRS N-Calls | rCRS N-Calls | N-calls decrease % | Total N-Calls | Total N-Calls | Total N-calls decrease % |
| --- | --- | --- | --- | --- | --- | --- |
|  | GSEQ | ResqMi |  | GSEQ | ResqMi |  |
| mtDNA1 | 631 | 307 | 51 | 6636 | 4674 | 30 |
| mtDNA2 | 783 | 376 | 52 | 7044 | 5025 | 29 |
| mtDNA3 | 630 | 291 | 54 | 6724 | 4552 | 32 |
| mtDNA4 | 792 | 530 | 33 | 7277 | 5293 | 27 |
| mtDNA5 | 607 | 336 | 45 | 7019 | 4981 | 29 |
| mtDNA6 | 812 | 529 | 35 | 7413 | 5344 | 28 |
| mtDNA7 | 373 | 349 | 6 | 6508 | 4483 | 31 |
| mtDNA8 | 681 | 725 | -6 | 9787 | 8147 | 17 |
| mtDNA9 | 658 | 424 | 36 | 8218 | 5770 | 30 |
| mtDNA10 | 565 | 384 | 32 | 7335 | 5370 | 27 |
| mtDNA11 | 659 | 436 | 34 | 7496 | 5664 | 24 |
| mtDNA12 | 550 | 366 | 33 | 6582 | 4945 | 25 |
| mtDNA13 | 550 | 367 | 33 | 6898 | 5050 | 27 |
| mtDNA14 | 587 | 359 | 39 | 7275 | 5632 | 23 |
| mtDNA15 | 505 | 337 | 33 | 6466 | 4998 | 23 |
| mtDNA16 | 580 | 348 | 40 | 6718 | 4609 | 31 |
| Mean | 623 | 407 | *35 | 7212 | 5284 | *27 |

**Table S3: Total sequence variants (homoplasmic and heteroplasmic) found in mtDNA sequences compared to rCRS using MitoChip v2.0.** The results from Affymetrix GSEQ are compared to sPROFILER and ResqMi software output. Sample names refer to DNA extracted from fibroblast (mtDNA1-8) and blood (mtDNA 9-16) samples.

| **Samples** | **Total sequence variants (rCRS)** | | |
| --- | --- | --- | --- |
|  | GSEQ | sPROFILER | ResqMi |
| mtDNA1 | 19 | 19 | 35 |
| mtDNA2 | 24 | 24 | 39 |
| mtDNA3 | 39 | 39 | 43 |
| mtDNA4 | 39 | 39 | 43 |
| mtDNA5 | 20 | 20 | 22 |
| mtDNA6 | 27 | 27 | 24 |
| mtDNA7 | 34 | 34 | 38 |
| mtDNA8 | 15 | 15 | 21 |
| mtDNA9 | 43 | 43 | 68 |
| mtDNA10 | 30 | 30 | 54 |
| mtDNA11 | 41 | 41 | 57 |
| mtDNA12 | 39 | 39 | 54 |
| mtDNA13 | 25 | 25 | 47 |
| mtDNA14 | 13 | 13 | 21 |
| mtDNA15 | 15 | 15 | 24 |
| mtDNA16 | 39 | 39 | 53 |

**
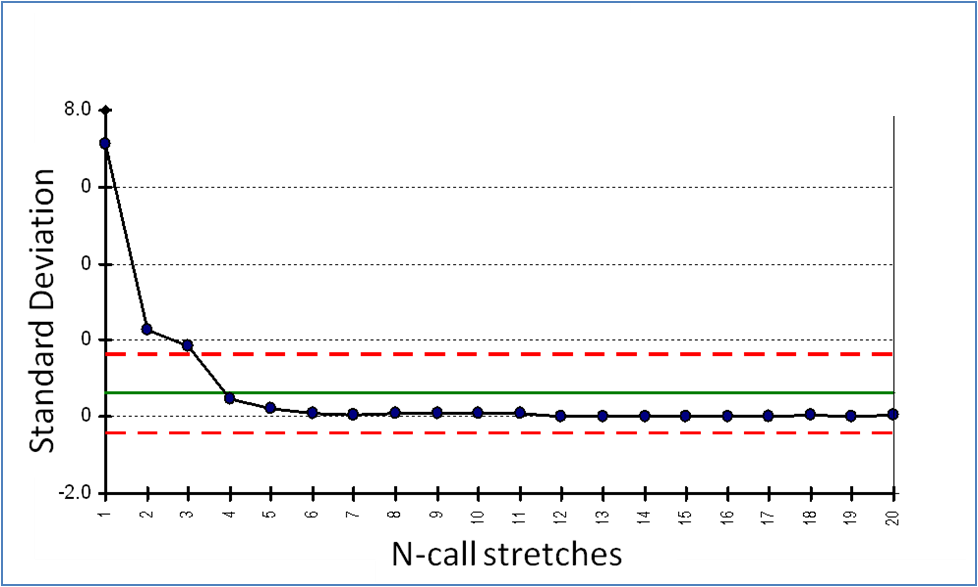
Figure S1: Control Chart calculation based on the standard deviation of the N-call stretches.** Shows that N-call stretches ≥ 4 bases long were within the limits.
